# Epithelial Remodeling and Epithelial Wavefront Aberrometry after Spherical vs. Cylindrical Myopic Small Incision Lenticule Extraction (SMILE)

**DOI:** 10.3390/jcm13133970

**Published:** 2024-07-07

**Authors:** Barbara S. Brunner, Lukas Feldhaus, Wolfgang J. Mayer, Jakob Siedlecki, Martin Dirisamer, Siegfried G. Priglinger, Stefan Kassumeh, Nikolaus Luft

**Affiliations:** 1 Department of Ophthalmology, LMU University Hospital, LMU Munich, 80539 Munich, Germany; 2 Auge Laser Chirurgie, 4020 Linz, Austria

**Keywords:** epithelial remodeling, myopia, astigmatism, refractive surgery, laser refractive surgery, small incision lenticule extraction (SMILE)

## Abstract

**Background/Objectives**: To compare the epithelial thickness changes and the changes in epithelial wavefront aberrometry following spherical versus astigmatic myopic small incision lenticule extraction (SMILE). **Methods**: Eighty-six eyes of 86 patients who underwent SMILE were included in this retrospective study. A total of 43 eyes underwent myopic spherical correction (spherical group) and 43 eyes underwent myopic cylindrical correction (cylindrical group). The groups were matched according to the spherical equivalent of surgically corrected refraction. Subjective manifest refraction as well as high-resolution anterior segment optical coherence tomography (MS-39; CSO; Florence, Italy) were obtained preoperatively as well as 3 months postoperatively. The latter was utilized for computing epithelial wavefront aberrometry in addition to epithelial thickness mapping. **Results:** Epithelial thickness increased significantly in both groups after SMILE (*p* < 0.01). In the cylindrical group, epithelial thickening was more pronounced on the flat meridian compared to the steep meridian (*p* = 0.04). In both groups, epithelial wavefront aberrometry showed a significant postoperative increase in the epithelium’s spherical refractive power, causing a myopization of −0.24 ± 0.42 diopters (D) in the spherical group (*p* < 0.01) and −0.41 ± 0.52 D in the cylindrical group (*p* < 0.0001). While no significant changes in epithelial cylindrical refractive power were observed in the spherical group, a significant increase was noted in the cylindrical group from −0.21 ± 0.24 D to −0.37 ± 0.31 D (*p* = 0.01). In both groups, epithelial higher-order aberrations increased significantly (*p* < 0.001). **Conclusions**: Postoperative epithelial remodeling after SMILE alters lower-order (sphere and cylinder) and higher-order aberrations of the corneal epithelial wavefront and might contribute to refractive undercorrection, especially in astigmatic corrections. Epithelial wavefront aberrometry can be used to quantify the refractive effect of epithelial remodeling processes after keratorefractive surgery.

## 1. Introduction

The concept of corneal epithelial remodeling was described by Alfred Vogt as early as 1921 in the form of focal epithelial thickening over an injury-related stromal depression [1]. According to this concept, the corneal epithelium will compensate for changes in the stromal surface curvature by modifying its thickness profile in an effort to maintain the integrity of the corneal optical surface [1]. Today, the following four laws of epithelial compensation have been formulized [2]. (1) The epithelium thickens in areas affected by tissue removal or flattening of the curvature. (2) The epithelium undergoes thinning in regions that are relatively elevated or in those areas of steepened curvature. (3) The extent of the epithelial changes correlates with the extent of the curvature change. (4) The magnitude of epithelial remodeling is defined by the rate of change in the curvature of an irregularity, meaning that small changes can be almost completely compensated by remodeling, while large changes can only be partially smoothed.

This compensatory potential of the corneal epithelium can be useful following stromal defects or curvature changes induced, e.g., by foreign body trauma, keratitis, or ectasia. In keratorefractive surgery, however, surgically induced corneal curvature change also elicits epithelial remodeling in proportion to the amount of surgically induced refractive correction [3,4]. Evidence has accumulated that these remodeling processes might play a role in postoperative myopic regression [5,6,7]. Similar epithelial compensatory mechanisms might also interfere with cylindrical keratorefractive corrections (i.e., asymmetric epithelial remodeling processes have been suspected) [8]. Indeed, Pedersen et al. [9] reported that myopic astigmatic small incision lenticule extraction (SMILE) with a preoperative cylinder of 0.75 to four diopters (D) resulted in an astigmatic undercorrection of approximately 11% per diopter of attempted correction. Moreover, Ivarsen et al. [10] compared cylindrical SMILE outcomes between low-astigmatism (<2.5 D) and high-astigmatism (≥2.5 D) treatments. While the first group exhibited an astigmatic undercorrection of approximately 13% per diopter of attempted correction, the average undercorrection in the second group was approximately 16% per diopter of attempted correction suggesting a dose-dependent remodeling of corneal (epithelial) tissue.

Today, high-frequency ultrasound as well as anterior segment optical coherence tomography (AS-OCT)-based epithelial thickness mapping allow for reliable morphological evaluation of the discussed compensatory epithelial remodeling processes after keratorefractive surgery [4,6,11]. Very recently, a novel AS-OCT-based imaging technique was introduced that allows for an additional optical wavefront analysis of the corneal epithelium called epithelial wavefront aberrometry. This novel technique might broaden our understanding of corneal epithelial power changes due to epithelial thickness remodeling after keratorefractive surgery by analyzing the spherical and cylindrical refractive power of the corneal epithelial layer as well its higher-order aberrometric profile [6]. Canto-Cerdan et al. proved the repeatability of this novel imaging technique with an intraclass correlation (ICCs) of >0.7 for epithelial refractive power measurements [6]. The same group previously employed epithelial wavefront aberrometry to compare the postoperative epithelial refractive remodeling between SMILE and femtosecond laser-assisted laser in situ keratomileusis (fs-LASIK), which amounted to a mean refractive regression of −0.53 D and −0.11 D of sphere, respectively [6].

In the present study, AS-OCT-based epithelial thickness mapping combined with epithelial wavefront aberrometry were used to analyze and compare not only morphological but also refractive epithelial remodeling between two subgroups of SMILE patients with purely spherical and cylindrical refractive error, respectively.

## 2. Materials and Methods

All patients included in this retrospective case series were treated at the Department of Ophthalmology, LMU University Hospital, LMU Munich, Munich, Germany. The study was approved by the local institutional review board of the Ludwig-Maximilians University (approval number: 23-0732) and adhered to the tenets outlined in the Declaration of Helsinki. Written informed consent to use their clinical data for statistical analysis was obtained from all participants.

### 2.1. Inclusion/Exclusion Criteria

All patients who underwent SMILE between 1 January 2022 and 1 August 2023 meeting the following inclusion criteria were included in the study. The inclusion criteria complied with the German national “Kommission Refraktive Chirurgie” (KRC) recommendations [12]. Patients with refractive myopia of up to −8.00 diopters (D) and/or refractive astigmatism of up to −5.00 D were included. In the spherical group, the maximum tolerated amount of preoperative refractive cylinder at the spectacle plane was 0.50 D. In the cylindrical group, the minimum required amount of preoperative refractive cylinder at the spectacle plane was 1.25 D. The minimal required preoperative corneal pachymetry was 480 µm. The programmed residual stromal thickness after keratorefractive lenticule extraction had to be at least 250 µm below the SMILE cap. All patients had to be at least 18 years old and show refractive stability (change <0.50 D over the 12 months prior to SMILE).

### 2.2. Pre- and Postoperative Measurements

All study participants were examined both preoperatively and 3 months postoperatively. The examinations included uncorrected distance visual acuity (UDVA) and corrected distance visual acuity (CDVA) using subjective manifest refraction based on the Jackson cross-cylinder method as well as a standard ETDRS visual acuity chart at 4 m distance. In addition, cycloplegic refraction was taken preoperatively. Furthermore, slit lamp biomicroscopy of the anterior segment as well as a dilated fundus exam were performed. To assess pre- and postoperative corneal tomography, epithelial thickness mapping, and corneal epithelial wavefront analysis, an anterior segment optical coherence tomography system with integrated Placido disc technology (MS-39; Costruzione Strumenti Oftalmici [C.S.O.], Florence, Italy) was used.

Corneal epithelial thickness was analyzed over the central 6.00 mm zone. In the spherical group, the epithelial thicknesses of the 3–6 mm zone of the nasal and the temporal quadrant were averaged (flat meridian), as well as the 3–6 mm zone of the superior and inferior quadrant (steep meridian). In the cylindrical group, epithelial thickness was assessed in the same fashion, when refractive with-the-rule (WTR) astigmatism (cylinder axis of 180 ± 20° in manifest refraction) was present. In eyes with refractive against-the-rule (ATR) astigmatism (cylinder axis of 90 ± 20° in manifest refraction), the average between the nasal and temporal 3–6 mm zone quadrants was considered to be the steep meridian and vice versa. The mean epithelial thicknesses of the steep and the flat meridian of the 3–6 mm zone were compared to each other, and their difference was calculated.

A built-in segmentation algorithm also enables the MS-39 to create up to an 8 mm epithelial thickness map. As Liu et al. already excluded the central peripheral 6–8 mm ring from the analysis in their study published in 2023 due to its low repeatability [13], the present study also exclusively analyzed the abovementioned reliable measurement zones.

Corneal wavefront aberrometry was analyzed with a virtual pupil diameter of 6.00 mm centered on the corneal vertex. The refractive index of the epithelium was defined as 1.401 [14]. The virtually refracted rays were finally used to calculate the epithelial wavefront error using the onboard software (Phoenix v.4.0, C.S.O., Florence, Italy). First, the optical wavefront of the epithelium is determined using the MS-39. Therefore, a light interaction with the epithelial layer and its interfaces, both with the air–tear layer interface and with the epithelial–stroma interface, is simulated, and the abovementioned optical wavefront is thus determined. A bundle of collimated beams with a given diameter is virtually projected onto the epithelium, deflected according to the corneal inclination, and finally deflected according to the Bowman layer inclination.

The computed term “Epi-Rx” is based on spherical (D), cylindrical (D), and axial (°) values and represents the lower-order aberrations of the epithelial wavefront. Moreover, the following corneal epithelial wavefront parameters were computed as root mean square (RMS) values: total wavefront error (“Epi-WFE”), higher-order aberrations (HOAs), spherical aberration, coma, and trefoil. The RMS wavefront error is defined as a key metric in the field of optical systems providing a quantitative measure of wavefront aberration. The RMS metric is a numerical indicator representing the accuracy between the actual wavefront of an optical system and a perfect spherical wavefront.

### 2.3. Surgical Procedure and Postoperative Therapy Regimen

All SMILE procedures were performed by a total of three experienced professionals (MD, WJM, NL) at a tertiary treatment center (Department of Ophthalmology, LMU University Hospital, LMU Munich, Munich, Germany). A 500 kHz femtosecond laser (VisuMax 500; Carl Zeiss Meditec AG, Jena, Germany) was used for the treatments. During all procedures, the same laser scanning settings (4.5 µm spot spacing for the cap and lenticule interface at 160 nJ laser energy) were maintained. A 4.00 mm incision was created at the 135° (right eyes) or 45° (left eyes) position. The optical zone was programmed to 6.4–6.7 mm, and the cap thickness was programmed between 120 and 140 µm. The postoperative therapy included the use of dexamethasone/tobramycin eye drops six times per day for one week, as well as dexamethasone eye drops four times a day beginning one week after the surgery and slowly tapered over the course of four weeks. Furthermore, preservative-free lacrimal eye drops were recommended as needed.

### 2.4. Data Management and Statistical Analysis

All data were gathered in Microsoft Excel spreadsheets (Version 16.82 for Mac; Microsoft, Redmond, WA, USA). Statistical analysis was performed using Prism 8 (GraphPad Software, San Diego, CA, USA). All results are expressed as mean ± standard deviation (SD) and range. To compare nominal-scale parameters (e.g., sex distribution) between groups, Fisher’s exact test was used. As the Kolmogorov–Smirnov test indicated non-normal distribution of the data, the Mann–Whitney U-test was performed to compare quantitative parameters (e.g., age) between groups. A *p*-value less than 0.05 was considered to be statistically significant.

## 3. Results

### 3.1. Patient Demographics and Baseline Parameters

A total of 86 eyes of 86 patients fulfilling the inclusion criteria were recruited and divided into a spherical SMILE group (43 eyes) and an astigmatic SMILE group (43 eyes). The mean preoperative manifest spherical equivalent was matched between groups and amounted to −3.81 ± 1.48 D in the spherical group and −3.81 ± 1.55 D in the cylindrical group (*p* = 0.85; Table 1). Mean age at the time of surgery was 32.9 ± 6.5 years in the spherical group and 33.2 ± 6.9 years in the cylindrical group (*p* = 0.82). As intended by the study design, there was a statistically significant difference in the refractive cylinder between the spherical (−0.17 ± 0.18 D) and the cylindrical group (−1.99 ± 0.78 D; *p* < 0.0001), respectively (Table 1). Further, BCDVA was significantly higher and K values significantly steeper in the spherical group preoperatively (Table 1).

### 3.2. Refractive Outcome

**Figure 1:**
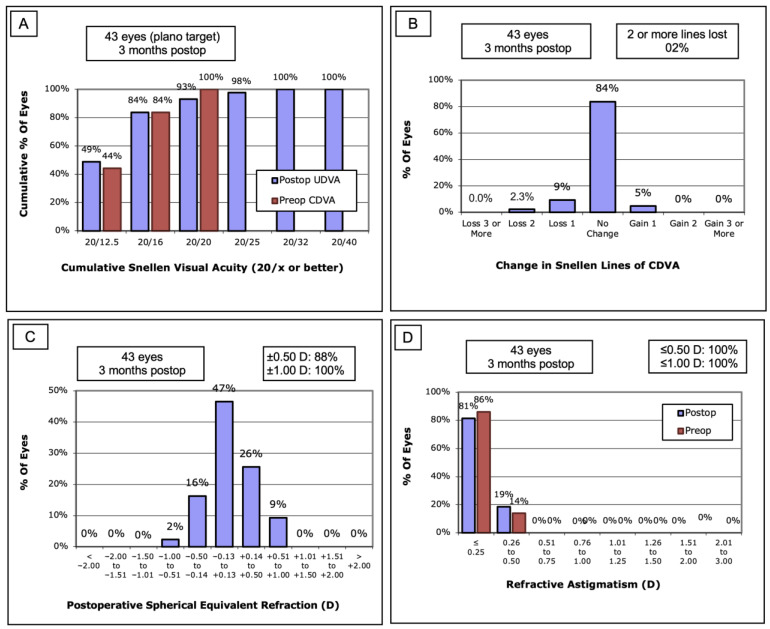
**Refractive outcome in the spherical myopic group after 3 months.** (**A**) Cumulative visual acuity. (**B**) Change in CDVA. (**C**) Postoperative spherical equivalent refraction. (**D**) Refractive astigmatism.

Figure 1 depicts the refractive outcome three months after spherical myopic SMILE. In total, 93% of the patients presented with an uncorrected distance visual acuity of 20/20 or better after treatment (Figure 1A). A loss of two lines in corrected distance visual acuity could only be observed in one patient (Figure 1B). A total of 88% of the patients were within a spherical equivalent of ±0.50 diopters, and 100% were within ±1.00 diopters (Figure 1C). As expected, refractive astigmatism was ≤0.50 diopters pre- and postoperatively in the spherical myopic group (Figure 1D).

**Figure 2:**
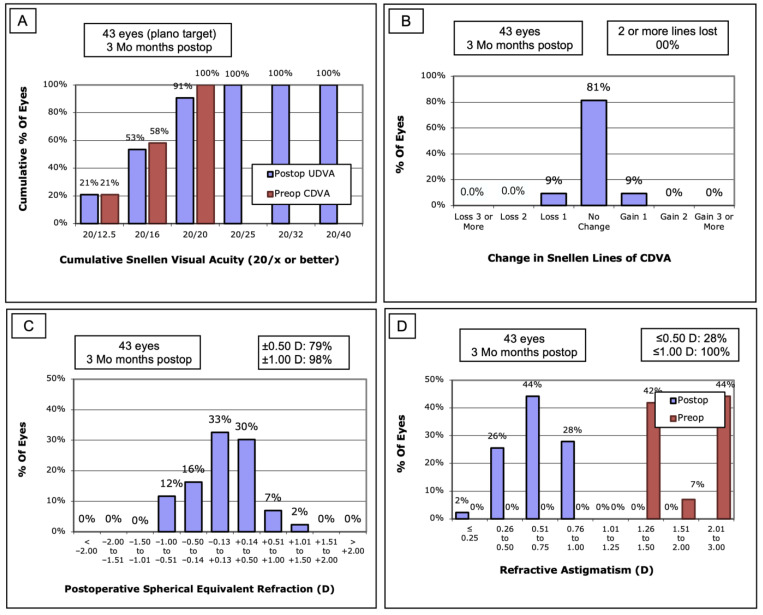
**Refractive outcome in the astigmatic myopic group after 3 months.** (**A**) Cumulative visual acuity. (**B**) Change in CDVA. (**C**) Postoperative spherical equivalent refraction. (**D**) Refractive astigmatism.

Figure 2 summarizes the refractive outcome three months after astigmatic myopic SMILE. In total, 91% of the patients achieved an uncorrected distance visual acuity of 20/20 or better after treatment (Figure 2A). A loss of one line in corrected distance visual acuity could only be observed in four patients, whereas none of the patients lost ≥ two lines (Figure 2B). A total of 79% of the patients were within a spherical equivalent of ±0.50 diopters, and 98% were within ±1.00 diopters (Figure 2C). The preoperative manifest cylinder is significantly higher in the astigmatic myopic smile group compared to the spherical myopic smile group. In total, 44% of the patients showed a preoperative manifest cylinder between 2.01 and 3.0 dpt. The manifest cylinder was significantly reduced three months postoperatively. In about 28% of the patients, the refractive astigmatism was ≤0.50 diopters. None of the patients showed a refractive astigmatism >1 diopter (Figure 2D).

### 3.3. Epithelial Thickness Changes (Remodeling)

**Figure 3:**
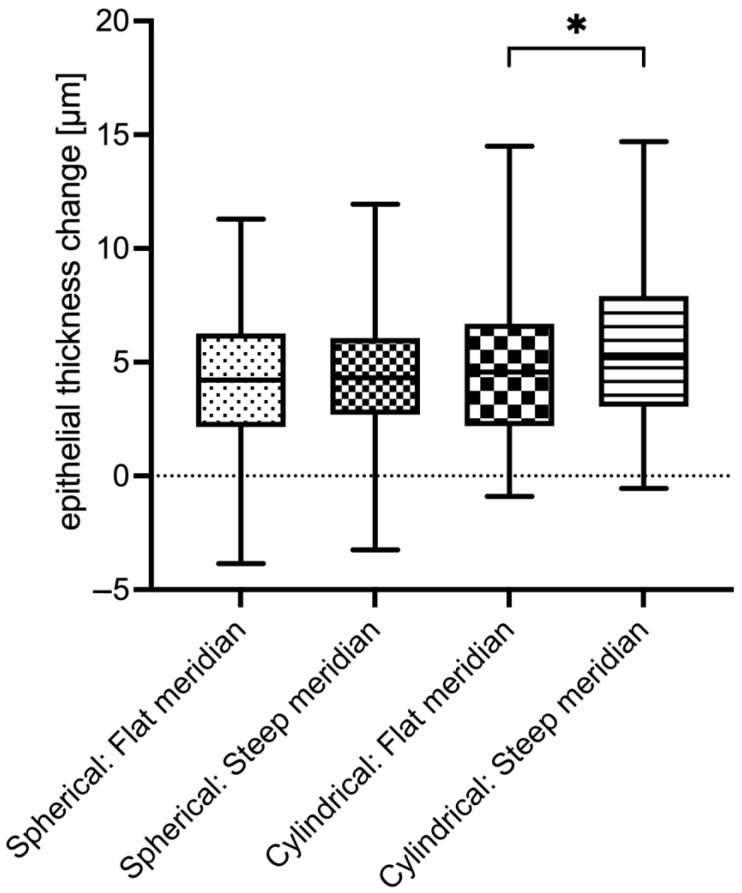
**Epithelial thickness changes 3 months after SMILE.** Depicted is the epithelial thickness change in the flat and steep meridian for both the spherical (*n* = 43) and the cylindrical group (*n* = 43). * *p* < 0.05.

In the spherical group, the preoperative epithelial thickness amounted to 53.9 ± 3.2 µm on the steep meridian versus 53.9 ± 3.3 µm on the flat meridian (*p* = 0.92), respectively. Three months postoperatively, the epithelial thickness increased homogeneously by 4.4 ± 3.5 µm on the steep meridian and 4.4 ± 3.2 on the flat meridian (both with *p* < 0.0001), with no statistically significant difference between the meridians (*p* = 0.91; Figure 3).

**Figure 4:**
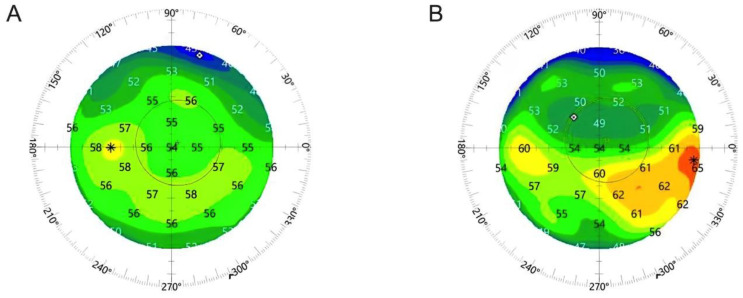
**Representative epithelial thickness map of an eye with myopia and with-the-rule astigmatism.** (**A**) Preoperatively and (**B**) three months postoperatively. An increase in the epithelial thickness majorly on the flat meridian (horizontal) can be observed.

In the cylindrical group, however, the epithelial thickness increased meridionally from 52.8 ± 3.9 µm to 57.3 ± 3.0 µm on the steep meridian (*p* < 0.0001) and from 53.1 ± 4.3 µm to 58.7 ± 3.3 µm on the flat meridian (*p* < 0.0001). This translated into a statistically significant meridional difference in epithelial thickening, which was more pronounced on the flat (5.6 ± 3.6 µm) than on the steep (4.5 ± 3.3 µm) meridian (*p* = 0.04; Figure 3). Preoperatively, there was no statistically significant difference between the epithelial thickness on the steep and flat meridian (*p* = 0.76) in the cylindrical group. A representative epithelial cell map of a high astigmatic eye (2.5 diopters on the corneal plane) before and after SMILE is displayed in Figure 4.

### 3.4. Epithelial Refractive Power Changes

**Figure 5:**
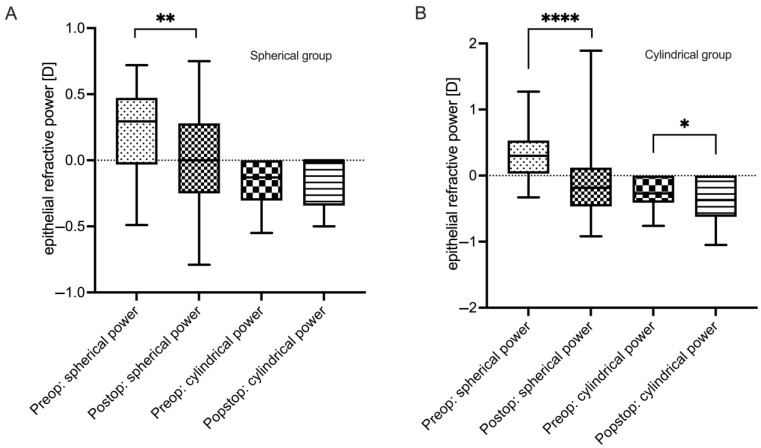
**Epithelial refractive power changes 3 months after SMILE.** (**A**) Spherical and cylindrical power pre- and postoperatively of the spherical group (*n* = 43). (**B**) Spherical and cylindrical power pre- and postoperatively in the cylindrical group (*n* = 43). * *p* < 0.05, ** *p* < 0.01, **** *p* < 0.0001.

In the spherical group, the refractive power of the epithelium amounted to 0.22 ± 0.32 D of the sphere before SMILE. Postoperatively, a significant change (i.e., myopization) of −0.24 ± 0.42 D (*p* = <0.01; Figure 5A) was observed. Similarly, in the cylindrical group, a statistically significant change from 0.29 ± 0.39 D to −0.13 ± 0.49 D of the epithelium’s refractive error—resulting in a myopization of −0.41 ± 0.52 D (*p* < 0.0001; Figure 5B)—was detected.

While no significant change in the epithelium’s cylindrical power could be observed in the spherical group (−0.18 ± 0.20 D preoperatively vs. −0.13 ± 0.19 D postoperatively; *p* = 0.26; Figure 5A), we detected a statistically significant increase in the cylindrical group (−0.21 ± 0.24 D preoperatively vs. −0.37 ± 0.31 postoperatively; *p* = 0.01; Figure 5B).

### 3.5. Epithelial Wavefront Error and Higher-Order Aberrations

**Figure 6:**
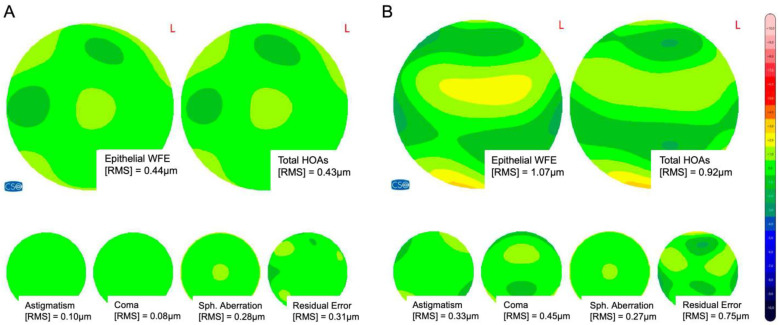
**Representative corneal epithelial wavefront aberrometry.** (**A**) Preoperatively and (**B**) three months after SMILE. WFE = wavefront error; HOAs = higher-order aberrations; RMS = root mean square.

In the spherical and cylindrical group, the total epithelial wavefront error (Epi-WFE) increased from 0.60 ± 0.25 µm to 0.74 ± 0.35 (*p* = 0.02; Table 2) and from 0.62 ± 0.22 µm to 0.92 ± 0.35 µm (*p* < 0.001; Table 2), respectively. Epithelial higher-order aberrations increased statistically significantly in the spherical group (from 0.37 ± 0.12 µm to 0.50 ± 0.18 µm; *p* < 0.0001) as well as in the cylindrical group (from 0.35 ± 0.12 µm to 0.53 ± 0.20 µm; *p* < 0.0001) (Table 2). We could also observe a statistically significant increase in the HOA terms spherical aberration and coma in both groups (Table 2). Interestingly, while trefoil increased significantly in the cylindrical group (*p* = 0.01), there was no change in the spherical group (*p* = 0.82; Table 2). Figure 6 displays a representative image of the corneal epithelial wavefront aberrometry of a left eye with with-the-rule astigmatism pre- and postoperatively (Figure 6).

## 4. Discussion

Myopic regression is a well-known phenomenon after keratorefractive surgery. As corneal imaging modalities have become more precise, epithelial compensatory mechanisms have turned out to be a major trigger of this early postoperative shift [15]. As early as 2012, Reinstein et al. [16] observed a postoperative lenticular change in the epithelial thickness profile, with greater thickening in the central areas, as part of a study on myopia correction using laser in situ keratomileusis (LASIK). The greatest epithelial thickness increase was observed within the first month after LASIK, whereas the epithelial thickness profile stabilized in the later follow-up period (3 months postoperatively to 12 months postoperatively). These findings are in line with a later study by Luft et al. on epithelial remodeling after SMILE, which showed an exponential recovery function of epithelial remodeling with the greatest thickening response within the first 24 h after surgery and a stabilization at 3 months postoperatively [4]. Zhu et al. confirmed that epithelial remodeling stabilizes 3 months after SMILE and femtosecond (fs-)LASIK, while in transepithelial photorefractive keratectomy (trans-PRK), it may take up to 6 months [17].

Furthermore, Liu et al. also conducted a study showing a significant increase in epithelial thickness in the early phase after SMILE, i.e., within the first postoperative month. The remodeling pattern varied in the different corneal sectors, with the greatest epithelial thickening being detected in the paracentral areas (3.9 μm over the 3 to 6 mm annulus) [13]. Similar results were obtained by Canto-Cerdan et al. [6] and Vinciguerra et al. [18]. Recently, Yang et al. found epithelial thickness changes of 4.01 ± 1.80 µm for FS-LASIK and 4.01 ± 2.03 µm for trans-PRK in the central 5 mm of the cornea, which were comparable to our absolute epithelial thickness changes following myopic SMILE. However, they included eyes with an astigmatism of up to diopters without subgroup analysis or consideration of the steep and flat meridian [19].

Very recently, Canto-Cerdan et al. were first to employ sophisticated epithelial imaging in form of epithelial wavefront aberrometry in their matched study of 77 SMILE and 77 fs-LASIK eyes. A mean myopic regression of −0.11 D after SMILE and −0.53 D after LASIK attributable to thickening-related remodeling of the epithelial refractive power was demonstrated within the first six postoperative months [6]. These findings are highly coherent with the present analysis showing an epithelium-related myopization of −0.24 D and −0.41 D in the spherical and cylindrical group, respectively, during the first 3 postoperative months. Of note, the spherical equivalent before SMILE was almost identical in both studies with −4.02 D and −3.81 D, respectively. Further research seems warranted to investigate the more pronounced spherical refractive regression after cylindrical compared with purely spherical SMILE observed in the present study.

Yu et al. postulated a higher tendency of epithelial thickness increase in the flat meridian following SMILE for myopic astigmatism [8]. Nevertheless, they did not compare data with a spherical control group. Additionally, both eyes of one patient were used. Therefore, this is the first comparative study to demonstrate meridional epithelial thickening changes in spherical and cylindrical myopic SMILE patients with a change in cylindrical epithelial power from −0.21 ± 0.24 D preoperatively to −0.37 ± 0.31 D at three months postoperatively. In contrast, no change in cylindrical corneal epithelial power was observed in the spherical SMILE group. Accordingly, epithelial thickness was homogeneous on the flat and steep meridian in purely spherical corrections. Of note, when cylindrical correction was targeted, a significantly higher epithelial thickening was observed on the flat meridian when compared to the steep meridian. Bearing in mind the laws of epithelial compensation [3], we attribute this phenomenon to the specific geometry of the SMILE lenticule in astigmatic corrections, which is horizontal oval-shaped in the case of WTR astigmatism and vertical oval-shaped in ATR astigmatism. Gatinel et al. explained in 2002 that the correction of astigmatism leads to a convex lenticule ablated on the steep meridian, while a constant thickness ablation is performed on the flat meridian. This results in a maximum ablation volume on the flat meridian [20]. As we mentioned before, corneal epithelial compensation and subsequent thickening is pronounced where the most stromal ablation occurred [2]. Thus, the tendency of a thicker epithelium on the flat meridian after SMILE is explained. This is in line with the findings by Yu et al. described above [8]. In contrast, Ye et al. described an asymmetric lenticule-like pattern of corneal epithelial remodeling after SMILE in a cohort of 64 eyes. However, they did not distinguish between spherical and cylindrical treatment profiles whilst including eyes with a cylindrical refraction of up to −2.50 diopters [21]. Recently, Li et al. also found axis-specific epithelial remodeling in a cohort of 42 eyes with high astigmatism (≥2 D). They concluded that epithelial remodeling was more pronounced on the steep meridian, which seems contrary to most of the studies cited before. This might be due to the small sample size or the fact that both eyes were used in half of the individuals. Further, the significant difference was found in the central (<2 mm) part of the cornea, which might be confounded due to the correction of high myopic astigmatism with a mean spherical equivalent of 6.89 ± 2.14 D [22]. Therefore, our results indicate that meridional epithelial remodeling after astigmatic SMILE might contribute to the well-established propensity to the cylindrical refractive undercorrection of SMILE. In line with previous works by Pedersen et al. [9] and Ivarsen et al. [10], we recommend a compensatory adjustment of the cylindrical treatment nomogram by increasing astigmatism correction by 10%.

We detected statistically significant increments in all the investigated HOA terms in both groups. One exemption was trefoil, which only increased statistically significantly in the cylindrical group; however, this was not to a clinically significant level.

It is well known that SMILE induces total corneal HOAs depending on the amount of refractive correction [13], and a previous study of our group has shown that subjective quality of vision might, therefore, be deteriorated compared with phakic IOL implantation, which induces less corneal HOAs [23]. Further, Liu et al. reported an increase in total corneal HOAs in the 6 mm zone after myopic SMILE and concluded that the magnitude of alteration correlates with the corrected spherical equivalent. In addition, they found a significant correlation between the increase in corneal epithelial thickness and total corneal HOAs [13]. However, they did not consider the epithelial HOAs, which increased significantly after both spherical and cylindrical myopic SMILE in our study. Further research is warranted to investigate potential implications of SMILE-related epithelial HOA induction for subjective quality of vision, especially in astigmatic corrections.

The present study is mainly limited by its retrospective nature. Furthermore, it should be highlighted that the study design did not allow for assessing gender-specific epithelial remodeling. For example, Liu et al. observed a significant association between gender and central as well as paracentral epithelial thickness increase at one month after SMILE. The results showed that there was significantly more epithelial thickening in female patients [13]. Moreover, Liu et al. were able to show that the preoperative epithelial status might act as a potential predictor for postoperative remodeling processes. To simplify matters, epithelial inhomogeneity was quantified as the coefficient of variation in epithelial thickness (CV) in the study. Patients exhibiting a thicker epithelial profile as well as a higher CV preoperatively tended to show less change in terms of epithelial thickness and CV postoperatively. Thus, the preexisting epithelial profile should be considered in further studies. Considering the significant differences between the groups preoperatively, we would like to state the following: the significantly lower BCDVA in the cylindrical group may be due to the inability to correct high astigmatism sufficiently with spectacles, and therefore the measured BCDVA tends to be lower than in purely spherical myopia. In line, K values showed higher (steeper) values in the spherical group, which is more common in higher myopia than in lower myopia with high astigmatism. The femtosecond laser used in this study (VisuMax 500) lacks a feature for cyclotorsion control, which might lead to the undercorrection of astigmatism [24]. Its newer brother, the novel VisuMax 800 with the onboard OcuLign^®^ cyclotorsion tracking system, showed significantly less total HOAs when compared to the VisuMax 500, probably due to this extra feature [25]. Therefore, we encourage further studies comparing corneal epithelial remodeling and aberrometry between both devices.

In summary, epithelial wavefront aberrometry represents a novel corneal epithelial imaging modality that can be used to quantify the refractive effect of epithelial remodeling processes after keratorefractive surgery. Our findings show that postoperative epithelial remodeling after SMILE alters lower-order (sphere and cylinder) and higher-order aberrations of the corneal epithelial wavefront and might contribute to not only spherical but also astigmatic undercorrection.

## Figures and Tables

**Table 1:** **Patient demographics and baseline parameters.** UCDVA = uncorrected distance visual acuity; BCDVA = best corrected distance visual acuity; FE = Fisher’s exact test; * *p* < 0.05, ** *p* < 0.01, *** *p* < 0.001, **** *p* < 0.0001. Displayed as mean ± SD, minimum and maximum in squared brackets.

Parameter	Spherical Group	Cylindrical Group	*p*-Value
Age [years]	32.9 ± 6.5 [19; 55]	33.2 ± 6.9 [21; 48]	0.82
Eyes [*n*]	43	43	
Patients [*n*]	43	43	
Sex [m:f]	20:23	20:23	>0.99 (FE)
UCDVA pre-SMILE [logMAR]	1.05 ± 0.30 [1.40; 0.40]	0.95 ± 0.37 [1.40; 0.40]	0.44
BCDVA pre-SMILE [logMAR]	−0.11 ± 0.08 [0; −0.20]	−0.06 ± 0.10 [0.40; −0.20]	<0.01 **
Refractive sphere [D]	−3.73 ± 1.52 [−1.50; −8.5]	−2.82 ± 1.64 [0.25; −6.50]	0.02 *
Refractive cylinder [D]	−0.17 ± 0.18 [0; −0.50]	−1.99 ± 0.78 [−1.25; −5.00]	<0.0001 ****
Spherical equivalent [D]	−3.81 ± 1.48 [−1.50; −8.50]	−3.81 ± 1.55 [−0.88; −7.63]	0.85
Stromal thickness pre-SMILE [µm]	490 ± 31 [434; 546]	480 ± 32 [432; 532]	0.20
Total pachymetry preop. [µm]	545 ± 31 [486; 599]	534 ± 33 [483; 590]	0.19
K_1_ preoperative	43.82 ± 1.38 [41.26; 47.80]	42.22 ± 1.46 [39.11; 45.98]	<0.0001 ****
K_2_ preoperative	44.49 ± 1.37 [41.73; 48.33]	43.73 ± 1.29 [40.16; 47.55]	0.0079 **
K_Mean_ preoperative	44.17 ± 1.38 [41.56; 48.07]	42.96 ± 1.35 [39.63; 46.75]	0.0003 ***

**Table 2:** **Epithelial wavefront error and higher-order aberrations.** WFE = wavefront error; HOAs = higher-order aberrations; SA = spherical aberration; CDVA = corrected distance visual acuity; * *p* < 0.05, **** *p* < 0.0001. Displayed as mean ± SD, minimum and maximum in squared brackets.

	Spherical Group (*n* = 43)			Cylindrical Group (*n* = 43)		
Parameter	Preop.	Postop.	*p*-Value	Preop.	Postop.	*p*-Value
Epi-WFE [RMS, µm]	0.60 ± 0.25 [0.26; 1.48]	0.74 ± 0.35 [0.28; 2.39]	0.02 *	0.62 ± 0.22 [0.28; 1.19]	0.92 ± 0.35 [0.40; 2.43]	<0.0001 ****
Epi-HOAs [RMS; µm]	0.37 ± 0.12 [0.20; 0.77]	0.50 ± 0.18 [0.20; 1.26]	<0.0001 ****	0.35 ± 0.12 [0.16; 0.60]	0.53 ± 0.20 [0.26; 1.01]	<0.0001 ****
Epi-SA [RMS; µm]	0.12 ± 0.08 [0.01; 0.32]	0.25 ± 0.15 [0.01; 0.64]	<0.0001 ****	0.12 ± 0.10 [0.00; 0.33]	0.24 ± 0.13 [0.06; 0.71]	<0.0001 ****
Epi-Coma [RMS; µm]	0.12 ± 0.10 [0.00; 0.62]	0.25 ± 0.19 [0.01; 1.10]	<0.0001 ****	0.15 ± 0.10 [0.03; 0.38]	0.23 ± 0.17 [0.01; 0.65]	0.01 *
Epi-Trefoil [RMS; µm]	0.14 ± 0.09 [0.02; 0.36]	0.14 ± 0.07 [0.02; 0.29]	0.83	0.11 ± 0.07 [0.01; 0.27]	0.18 ± 0.14 [0.02; 0.61]	0.01 *

## Data Availability

The raw data supporting the conclusions of this article will be made available by the authors on request.
